# Influence of initial gas concentration on methane–air mixtures explosion characteristics and implications for safety management

**DOI:** 10.1038/s41598-023-40383-3

**Published:** 2023-08-19

**Authors:** Quansheng Jia, Rongjun Si, Lei Wang, Zhongbei Li, Shaoqian Xue

**Affiliations:** 1 China Coal Technology and Engineering Group Chongqing Research Institute, Chongqing, 400037 China; 2 Petroleum, Oil & Lubricants Department in Army Logistics Academy of PLA, Chongqing, 401331 China; 3 School of Civil, Mining, Environmental and Architectural Engineering, University of Wollongong, Wollongong, NSW 2522 Australia

**Keywords:** Engineering, Fossil fuels

## Abstract

Gas explosions, particularly those involving methane–air mixtures, present considerable hazards in confined spaces, such as coal mines. Comprehending the explosion characteristics and their correlations with initial gas concentrations is vital for devising effective safety measures. This study examines the influence of initial gas concentration on explosion temperature, overpressure, and flame evolution in methane–air premixed gas explosions, utilizing a custom-built 20-L spherical explosion experimental apparatus. The explosion temperatures display an oscillatory pattern, reaching maximum values at 6.5%, 9.5%, and 12% initial gas concentrations, with corresponding temperatures of 995 K, 932 K, and 1153 K. The maximum overpressure exhibits an initial rise and fall trend, modeled by an exponential function. Notably, in proximity to the 9.5% concentration, the pressure wave fosters the reverse propagation of the flame wave, leading to a secondary temperature increase. Flame sensors were employed to investigate the presence, absence, and duration of flames, demonstrating that elevated initial gas concentrations resulted in more prolonged flame durations and increased harm. At an initial gas concentration of 9.5%, a persistent flame is generated instantaneously during the explosion. Furthermore, the study analyzes the interplay between temperature and overpressure, underscoring the significance of mitigating high-temperature burns near tunnel walls and enclosed spaces. These findings advance the understanding of gas explosion dynamics and hold substantial implications for safety measures in coal mines.

## Introduction

Methane gas explosions are a major hazard in coal mines, leading to significant consequences, including economic losses for coal enterprises, human casualties, environmental damage, and severely hindering the coal industry's production^1,2^. Gas explosions release an immense amount of energy instantaneously, resulting in a high-temperature environment. In underground coal mines, explosions typically occur in excavation tunnels and mining faces, where environmental factors prevent the easy dissipation of explosion energy, causing sustained high temperatures in the tunnels and posing considerable risks to personnel and equipment^3–7^. The initial methane concentration influences the peak temperature and duration of the explosion. Investigating the effects of varying initial methane concentrations on temperature characteristics during the explosion process can help provide a crucial theoretical basis for understanding temperature characteristics in coal mine methane explosions, thus preventing the risks posed by gas explosions^8–11^.

In 1967, Olsen^12^ first derived an expression for explosion temperature through theoretical research. Scholars worldwide now use numerical simulation software such as FLACS^13,14^, FLUENT^15,16^, AutoReaGas^17,18^, and CHEMKIN^19^ to study methane explosion temperatures or establish specific mathematical physics equations to explore temperature variation rules under fixed volume or pipeline propagation conditions^20^. Some researchers have also simulated temperature variation rules for methane explosions in confined spaces, laying the groundwork for methane explosion temperature studies^21^. However, most simulations are conducted under isothermal or adiabatic conditions, leading to discrepancies with actual experimental data and preventing accurate simulations of temperature changes during real methane explosions.

Under experimental conditions, Wang and He^22^ used voltage signals to represent temperature, unveiling the temperature change trend of methane explosions as they propagate through pipelines. Subsequent researchers have studied temperature variations at different positions during pipeline propagation, finding that the flame temperature in the upper part of the pipeline is higher than in the lower part^23,24^. Cui et al.^25^ used R-type micro-thermocouples to examine the temperature variations of small-scale pipeline methane explosions. Li et al.^26^ employed C2-7-K and C2-1-K thermocouples to investigate temperature variations during explosion propagation, with the highest recorded temperature reaching 1292.27 K. Liu et al.^27^ analyzed the relationship between flame propagation and temperature during pipeline explosions, discovering that increased temperatures promote flame propagation. Nie et al.^28^ employed a two-dimensional temperature field radiation method to study temperature variations surrounding the explosion flame, finding that the temperature at the flame front initially rises sharply, then slows, and finally decreases after reaching its peak.

Explosion pressure under different initial methane concentrations is the primary parameter for studying methane explosions. Currently, due to their explosive nature, methane explosion experiments are mainly conducted in sealed containers. Pekalski et al.^29^ found that increasing ambient temperature reduces the maximum explosion pressure of CH_4_, while the maximum pressure rise rate does not change significantly. Shi et al.^30^ used a 20 L spherical explosion vessel to study the explosion characteristics and influencing factors of CH_4_-coal dust mixed explosions, finding that under equivalent concentration conditions with an initial pressure equal to atmospheric pressure for coal dust and CH_4_, the greater the initial pressure of the combustible gas, the higher the maximum explosion pressure and pressure rise rate. Shirvill et al.^31^ found that adding less than 25% H_2_ by volume to the gas network does not significantly increase the intensity of methane explosions. Jiao et al.^32^ established a relationship between overpressure and temperature during tunnel explosions, obtaining a relatively reliable distribution of air temperature in the tunnel as a function of propagation distance shortly after the explosion. This provided a direction for studying the relationship between temperature and pressure during the explosion process. Tran et al.^33^ used ANSYS Fluent to create a three-dimensional cylindrical geometric model, simulating the characteristics of CH_4_ explosions and concluding that the maximum explosion pressure occurs when the equivalence ratio of the CH_4_-air mixture is 1.2, and adding H_2_ to the fuel mixture will increase the maximum explosion pressure.

In summary, current research on temperature characteristics during methane explosions primarily focuses on numerical simulations, with relatively fewer studies on explosion temperature characteristics and the interactions between temperature and pressure under different initial methane concentrations. To address this, this paper experimentally investigates the temperature variations during the explosion process and the interactions between temperature and pressure under different initial methane concentrations. The findings contribute to the theoretical understanding of the mechanisms behind temperature variations during methane explosions, thereby enhancing strategies for gas explosion disaster prevention and control. Furthermore, a deeper comprehension of the interaction mechanisms between temperature, pressure, and flame during a methane explosion can provide a theoretical foundation for planning escape and rescue operations during gas explosion incidents.

## Methods

### Experiment system composition

**Figure 1:**
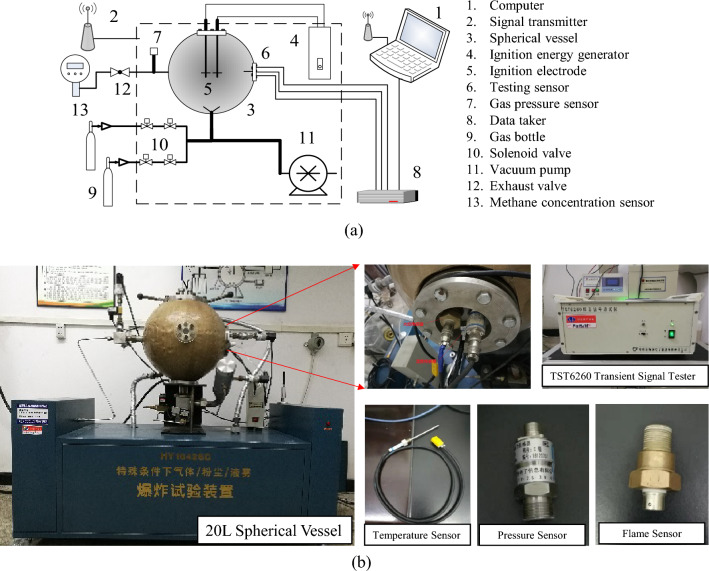
20 L Spherical explosion test system. (**a**) Schematic diagram. (**b**) apparatus setup.

The explosion experimental system comprises three primary modules: a 20 L spherical explosion module, a gas supply and distribution module, and a data acquisition module, as illustrated in Fig. 1. The experiment is conducted within a 20 L spherical explosion system utilizing an electric spark ignition with an energy of 10 J. The ignition signal is transmitted to the ignition energy generator via a computer and a signal transmitter, which subsequently detonates the gas mixture. This apparatus is specifically designed to study the explosive properties of gases concerning temperature, pressure, and flame evolution under controlled conditions. The device features a spherical vessel containing a gas mixture that, when ignited, triggers an explosion. The vessel's spherical shape allows for a uniform distribution of pressure and temperature during the explosion, making it an ideal tool for examining the effects of gas explosions.

The 20 L spherical explosion module is constructed with a stainless-steel material, and the vessel is designed to withstand a pressure of 4 MPa with a thickness of 20 mm. This design ensures the necessary strength and integrity of the vessel during the experiment.

The gas supply and distribution module primarily consist of solenoid valves, gas distribution pressure sensors, and laser CH_4_ concentration sensors. The CH_4_ concentration is initially estimated using the pressure proportioning method and is then fine-tuned with the aid of laser CH_4_ concentration sensors. These sensors can accurately calibrate the CH_4_ concentration within the vessel with a precision of 0.1%.

The data acquisition module comprises testing sensors and a transient signal test system. The testing sensors include temperature, pressure, and flame sensors, as shown in Fig. 1b. The temperature sensor is positioned 103 mm from the spherical vessel wall, with an accuracy of 0.1 K, and a frequency response of 5 kHz. Its response time is in the millisecond range, ensuring rapid and timely readings. With a measurement range of 273–2373 K, this sensor is capable of providing precise temperature measurements during the explosion. The pressure sensor is a high-frequency dynamic pressure sensor of the CYG400 series (inherent frequency 200 kHz), which accurately determines the pressure signal throughout the explosion process and measures the pressure within the vessel. Its measurement range is from 0 to 2 MPa, and all pressure measurements taken are relative pressures. The flame sensor is a CKG100 type with a response time of ≤ 10 μs, capable of capturing the flame within the explosion vessel and utilizing the flame signal collection channel as an internal trigger channel. Upon receiving the flame signal, which is when the testing system receives a high-level signal, the sensor triggers the temperature, pressure, and flame sensors to simultaneously collect, analyze, and store experimental data in the explosion vessel after processing by the transient signal test system. It's important to note that the temperature, pressure, and flame sensors used in our experiments were factory-calibrated prior to use. This ensures the reliability and accuracy of our measurements, providing a solid foundation for our data analysis.

### Experiment process

Initially, the spherical vessel was evacuated to 0.05 MPa. Subsequently, the required amount of CH_4_ gas was introduced into the vessel using the partial pressure proportioning method. A compressor was employed to inject air, ensuring the CH_4_/air mixture pressure reached 0.15 MPa. Following a 15-min resting period, the exhaust hole of the vessel was connected to a gas flow meter and a laser CH_4_ concentration sensor. The gas flow rate was adjusted until it stabilized at 200–300 mL/min, and the CH_4_ concentration was measured after 2 min. By adding air, the CH_4_ concentration in the vessel was fine-tuned until it reached the desired experimental value. The excess gas mixture was discharged to maintain the pressure inside the vessel at 0.12 MPa. It is important to note that the laser CH_4_ concentration sensor was calibrated using standard gas to ensure measurement accuracy of 0.1%.

Upon preparing the experiment, the trigger switch was activated, sequentially triggering the solenoid valve, high-pressure pulse generator, and data acquisition instrument to collect temperature, pressure, and flame data from the explosion vessel. The ignition electrode was then employed to ignite the gas combustion, initiating the explosion process.

Following each experimental set, combustion products were discharged, and the data was recorded and saved. Additionally, the recorded data underwent automatic filtering and noise reduction processing to enhance its accuracy and reliability. The gas in the vessel was replaced 2 to 3 times, and subsequent experiments were conducted after the temperature in the vessel returned to room temperature. The initial conditions for the experiments were room temperature, 0.12 MPa, and an experimental step of 0.5%. It is well-known that the lower explosion limit (LEL) and upper explosion limit (UEL) of a methane–air mixture are 5.0% and 16.0% respectively. In this study, we specifically focused on testing concentrations within the range of 5.5% to 14.0%.

## Results and discussion

### Explosion temperature

#### Temperature variation during the explosion process

**Figure 2:**
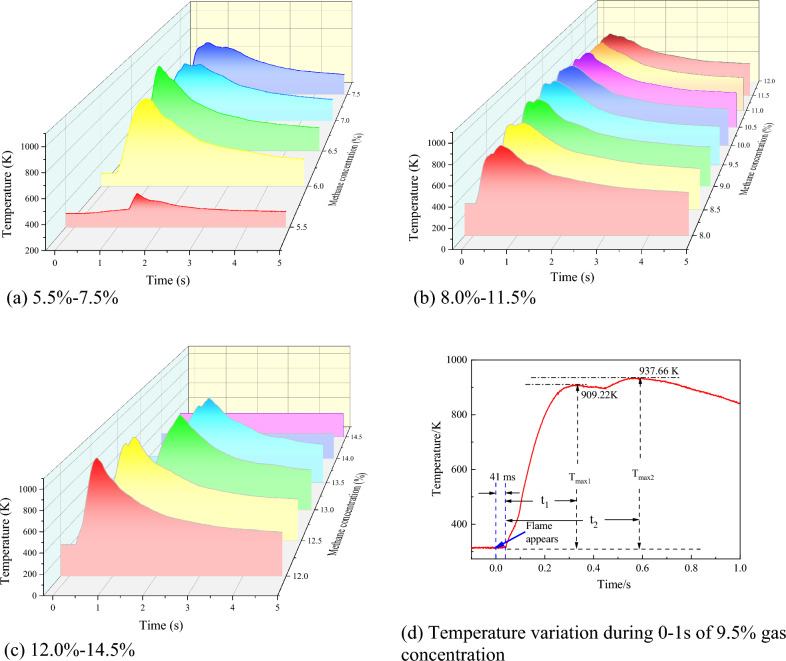
Temperature variation during gas explosion under different initial gas concentration conditions.

Gas explosions generate high temperatures that can lead to devastating consequences^34^. In this study, the relationship between explosion temperature and initial gas concentration was experimentally investigated, as illustrated in Fig. 2. As anticipated, both the rate of temperature change and peak explosion temperature varied depending on the initial gas concentration. A noticeable secondary temperature rise was observed during the explosion, particularly at concentrations near 9.5%, as shown in Fig. 2b.

Following ignition, there was a 41 ms delay before the temperature increased sharply. The first peak temperature of 909.22 K was reached at 331 ms, then decreased to 894.35 K at 450 ms, followed by a second rise to a peak of 937.66 K at 570 ms, and subsequently declined gradually. This phenomenon can be attributed to the explosion temperature test sensor being positioned 103 mm from the center of the vessel. The confining effect of the explosion vessel and pressure, along with other factors, cause the flame in the 20 L vessel to oscillate and superimpose, resulting in the secondary temperature rise. The enclosed space, such as the vessel wall, exerts a restraining effect on the explosion, emphasizing the importance of preventing high-temperature burns near tunnel walls and closed ends in actual gas explosion scenarios.

#### Maximum explosion temperature

**Figure 3:**
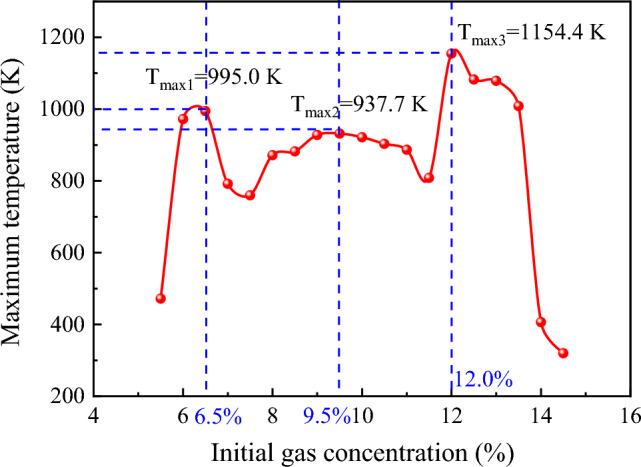
Maximum temperature of gas explosion under different initial gas concentration conditions.

The maximum explosion temperature refers to the highest temperature reached during an explosion at a specific gas concentration. Figure 3 presents the results of an investigation into the variations of maximum explosion temperatures for different initial gas concentrations within the explosion limit. The results reveal an oscillatory phenomenon of the maximum explosion temperature concerning the initial gas concentration within the explosion limit. The trend displays an increase–decrease-increase–decrease-increase–decrease pattern as the initial gas concentration increases. Notably, extreme values are observed at 6.5%, 9.5%, and 12% initial gas concentrations, with the highest explosion temperature of 1154.4 K attained at 12%.

The observed variation in the maximum explosion temperature with changes in the initial gas concentration can be attributed to the nature of the energy released during the gas explosion. The energy release during the gas explosion primarily occurs in the form of gas expansion and heat^35^. In the current experiment, the energy predominantly exists in high-pressure expansion gas, high-temperature environment, reaction products, and heat transfer. The energy of the reaction before the explosion varies with the initial gas concentration, and the high-temperature environment generated during the explosion represents one of the ways energy is released from the explosion process.

Figure 3 reveals that the highest explosion pressure is generated at a 9.5% gas concentration, indicating that the explosion process in the form of gas expansion releases more energy than at 6% and 12% gas concentrations. This results in slightly lower temperatures at equivalent gas concentrations compared to the latter two. For an initial gas concentration of 12%, the amount of methane involved in the reaction leads to a higher total amount of energy released during the explosion compared to 6%, resulting in higher stored energy in the form of temperature during the 12% gas explosion process. Furthermore, due to the influence of different gas explosion process flame durations, the highest explosion temperature exhibits a complex oscillation pattern with changes in the initial gas concentration.

### Explosion overpressure

**Figure 4:**
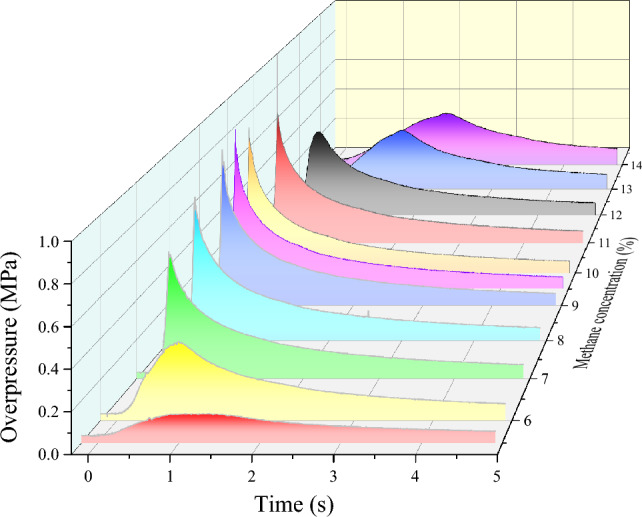
Overpressure evolution during gas explosion under different initial gas concentration conditions.

**Figure 5:**
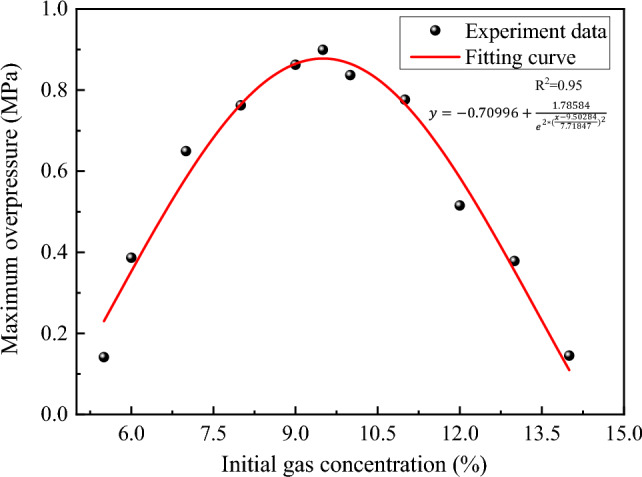
Maximum overpressure of gas explosion under different initial gas concentration conditions.

The evolution of overpressure during gas explosions was investigated under varying initial gas concentration conditions, and the results are presented in Fig. 4. It can be observed that the overpressure experienced during the explosion under different initial gas concentration conditions follows a similar trend, in which it initially increases and subsequently decreases. However, it was noted that the process of pressure rise and fall during the gas explosion near 9.5% initial gas concentrations occurred more rapidly. Figure 5 displays the maximum explosion overpressure and the corresponding time at different initial gas concentrations ranging from 5.5 to 14.5%. The maximum explosion pressure is observed to decrease as the initial gas concentration increases from 5.5 to 6.5%, with a slight increase at 7.0% followed by a gradual increase up to 9.5%, which reaches the peak value of 0.899 MPa. Subsequently, there is a decrease in the maximum explosion pressure at 10.0% and then an oscillating pattern between 10.5 and 13.5%, with a sharp decrease at 14.0% and 14.5%. The maximum overpressure appears to fit the equation $y=-0.70996+\frac{1.78584}{{e}^{2*{(\frac{x-9.50284}{7.71847})}^{2}}}$, with the fitting coefficient R^2^ > 0.9.

### Flame evolution

**Figure 6:**
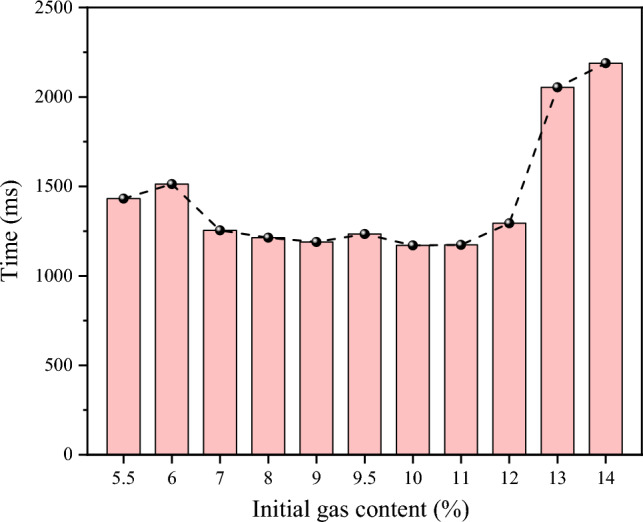
Flame duration of gas explosion under different initial gas concentration conditions.

Flame signals can be collected through a flame sensor, which detects voltage signals. The presence of a voltage signal indicates the existence of a flame. The voltage signal is utilized to study the presence, absence, and duration of the flame. As shown in (Fig. 6), the flame duration during explosions varies for different initial gas concentrations. The shortest flame durations are observed when the initial gas concentrations are at 9%, 10%, and 11%, with the duration remaining relatively constant across these concentrations. In contrast, longer flame durations are observed when the initial gas concentrations are at the UEL and LEL, which correspond to higher temperatures in the tank. Moreover, the flame duration during the explosion differs from that at 9.5% concentrations. Specifically, when the initial gas concentration is near the upper explosion limit, the flame duration during the explosion can be up to 1.84 times longer than at a 9.5% concentration

**Figure 7:**
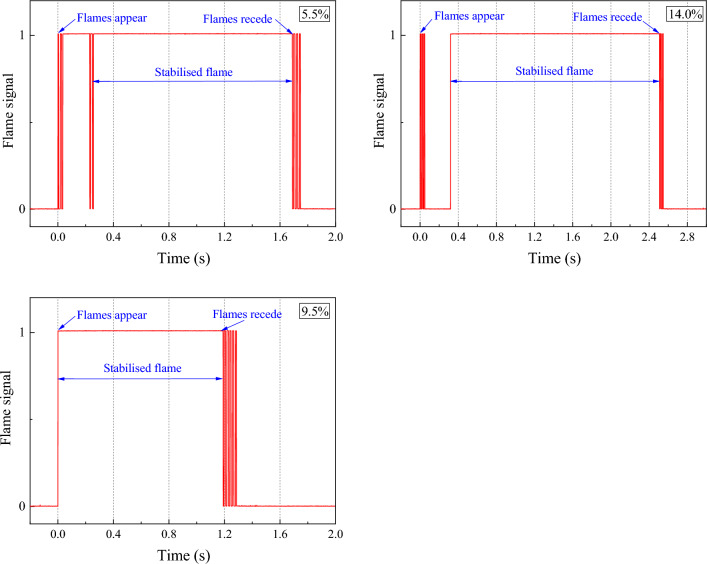
Explosion flame signals for 5.5%, 9.5% and 14.0% methane concentration.

When the initial gas concentration is near 9.5%, the explosion occurs at the same time as the flame signal is picked up, near the UEL and LEL, the flame sensor picks up the flame signal after a certain period of time before a stable explosion flame exists. The signal image of a typical flame is taken to study the change in flame during the explosion of low concentration gas (5.5%), equivalent concentration gas (9.5%) and high concentration gas (14%), as shown in Fig. 7. It was found that when the initial gas concentration was at 9.5%, the explosion occurred at the same time as the flame signal was picked up. However, near the UEL and LEL, the flame sensor picked up the flame signal, and a certain amount of time elapsed before a stable explosion flame was present.

When the initial gas concentration is near 9.5%, the explosion occurs simultaneously with the flame signal detection. Near the UEL and LEL, the flame sensor detects the flame signal after a certain period of time before a stable explosion flame exists. Figure 7 shows a typical flame signal image for studying the changes in flame during explosions of low concentration gas (5.5%), equivalent concentration gas (9.5%), and high concentration gas (14%).

Analysis of the flame signal images revealed that at an initial gas concentration of 5.5%, intermittent flames first appear in the vessel after ignition, indicating that the low gas concentration causes a weak initial explosion with an unstable flame. However, after a period, the explosion temperature and pressure rise, resulting in a stable explosion with a flame stability duration of 1432 ms. At an initial gas concentration of 9.5%, the explosion is more intense and the flame appears immediately after ignition, with a stable flame duration of 1232 ms. Conversely, at an initial gas concentration of 14%, the high gas concentration makes ignition difficult, causing the gas around the ignition source to ignite first and, after a period, produce a stable flame with a flame stability duration of 2189 ms.

**Figure 8:**
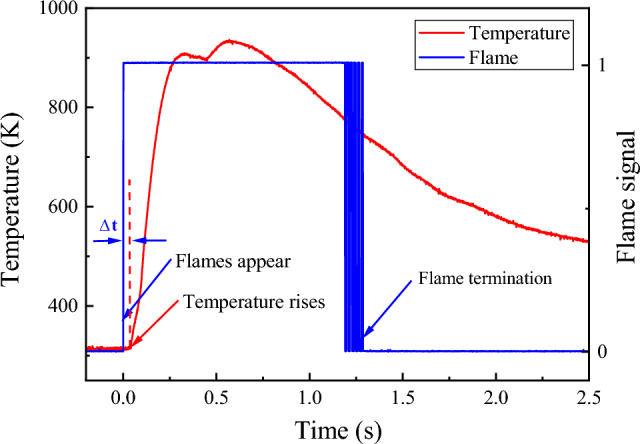
The relationship between temperature and flame during the explosion of 9.5% gas concentration.

Figure 8 illustrates the correlation between the explosion flame and the explosion temperature for a gas explosion at an initial gas concentration of 9.5%. It is evident that when the flame signal appears after a certain time interval (∆t), the temperature in the vessel begins to increase, consistent with the previously discussed temperature delay phenomenon. The highest temperature during the explosion is reached while the flame is present in the vessel. Subsequently, the flame extinguishes once the temperature in the tank drops to 750 K.

The analysis suggests that high initial gas concentrations result in longer flame durations during explosions, leading to greater harm to individuals. In addition to the toxic effects of post-explosion gases and the possibility of secondary explosions, the prolonged burning effect on the human body can cause severe damage to personnel. Therefore, in coal mines, preventing high concentrations of gas explosions is crucial for mitigating the hazards posed by high temperatures. Implementing measures to monitor and regulate gas concentrations in mines can help reduce the likelihood of gas explosions and ensure the safety of miners.

### The relationship between temperature and overpressure during gas explosion

**Figure 9:**
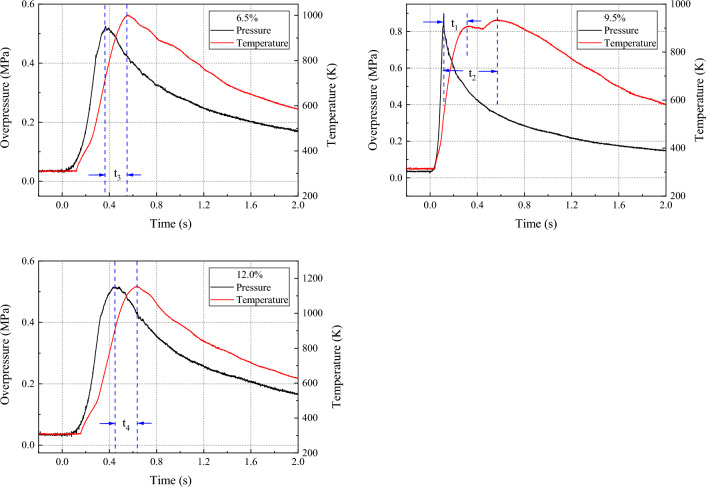
The relationship between overpressure and temperature under 6.5%, 9.5% and 12.0% gas concentrations.

Understanding the relationship between temperature and overpressure during gas explosions is crucial for comprehending the dynamics of the explosion process and developing effective safety measures. In this context, the temperature and overpressure of a 9.5% concentration gas explosion were analyzed, as illustrated in Fig. 9. The results show that the pressure peaked at 0.899 MPa for 115 ms, while the temperature reached its first peak at 909.22 K for 331 ms, with a pressure peak time interval of t_1_ = 216 ms. Additionally, the temperature peaked for the second time at 936.34 K for 570 ms, with a pressure peak time interval of t_2_ = 455 ms. Notably, the analysis demonstrates that the pressure wave propagated faster than the flame during the explosion process, causing the temperature to reach its first peak later than the pressure wave peak time.

Furthermore, as the explosion pressure wave reaches the vessel wall, it encounters a reverse force, resulting in its reverse propagation. Simultaneously, the slower burning wave converges with the reverse-propagating wave, causing some of the flame burning waves to also propagate in the opposite direction. The superposition of the forward and reverse burning waves leads to a further increase in the flame temperature. It is worth noting that this phenomenon can be hazardous, especially when the explosion strength is high, as it can result in a secondary increase in the gas explosion temperature.

However, when the initial gas concentration is near the UEL and LEL, for example at 6.5% and 12%, as shown in Fig. 9, no secondary increase in temperature during the explosion is observed. It is postulated that the reduced intensity of the explosion results in a slower pressure rise rate and a longer time for the pressure to reach its peak. Consequently, the ability of the pressure wave to reverse propagate upon reaching the explosion tank is weakened. Additionally, the time intervals between temperature peaks and pressure peaks, t_3_ and t_4_, are significantly shorter than t_2_, thereby diminishing the reverse propagation of the flame wave. As a result, the phenomenon of secondary heating during the temperature rise process is weakened, and in some cases, non-existent.

## Conclusion

This study has investigated the explosion characteristics of methane–air premixed gas and the influence of initial gas concentration on explosion temperature, overpressure, and flame evolution. The findings provide valuable insights into the dynamics of gas explosions and have significant implications for the development of effective safety measures. The main conclusions of this study are as follows:

1. The explosion characteristics of methane–air premixed gas are strongly influenced by the initial gas concentration. The explosion temperature exhibits an oscillatory pattern with increasing gas concentration, with maximum values occurring at 12% and 6.5% concentrations. The maximum overpressure follows an initial rise and fall trend and can be modeled with a fitting equation $y=-0.70996+\frac{1.78584}{{e}^{2*{(\frac{x-9.50284}{7.71847})}^{2}}}$, yielding a strong coefficient of determination (R^2^ > 0.9).
2. The relationship between temperature and overpressure during gas explosions reveals the causes of temperature changes. Pressure wave propagation is faster than flame propagation, resulting in a secondary increase in temperature during strong explosions. Near the 9.5% gas concentration, the explosion temperature exhibits two peaks over time. However, when the initial gas concentration is near the UEL and LEL, the phenomenon of secondary heating is weakened or non-existent. This highlights the importance of preventing high-temperature burns near tunnel walls and closed spaces in actual gas explosion scenarios.
3. Flame sensors were employed to study the presence, absence, and duration of flames, revealing that high initial gas concentrations led to longer flame durations and greater harm. Therefore, monitoring and regulating gas concentrations in potentially explosive environments, such as coal mines, are essential for mitigating hazards posed by gas explosions and ensuring personnel safety.
4. The explosion intensity near the explosion limits is reduced due to the influence of the initial gas concentration, leading to a slower rate of pressure rise during the explosion process, a longer time to reach the pressure peak, and a weakened ability of pressure waves to reverse propagate after reaching the explosion tank. The time intervals between pressure and temperature peaks during the explosion process are smaller, which diminishes the reverse propagation of the flame wave and weakens secondary heating during the temperature rise process, or even renders it non-existent. Methane–air premixed gas with a 9.5% methane concentration exhibits the most intense explosion effect, generating stronger pressure waves and a higher flame propagation speed.

## Data Availability

The datasets generated and analyzed during the current study are available from the corresponding author on reasonable request.
